# A Rare Case of Sunitinib-Induced Rhabdomyolysis in Renal Cell Carcinoma

**DOI:** 10.1155/2018/3808523

**Published:** 2018-07-19

**Authors:** Andrew D. Liman, Vida A. Passero, Agnes K. Liman, Jenna Shields

**Affiliations:** ^1^Hematology and Oncology, VA Pittsburgh Healthcare System, University of Pittsburgh School of Medicine, Pittsburgh, PA 15240, USA; ^2^Laboratory and Pathology, VA Pittsburgh Healthcare System, University of Pittsburgh School of Medicine, Pittsburgh, PA 15240, USA; ^3^Pharmacy, VA Pittsburgh Healthcare System, Pittsburgh, PA 15240, USA

## Abstract

We report a rare case of metastatic renal cell carcinoma (RCC) in a patient who developed rhabdomyolysis while on sunitinib. He was admitted to the hospital due to muscle weakness, fatigue, poor oral intake, and difficulty swallowing in March 2017. He was found to have pancytopenia, liver failure, kidney failure, high uric acid, and increased creatine phosphokinase of more than 5000. He quickly developed lactic acidosis and acute respiratory failure. He was transferred to the ICU, but his condition declined rapidly. He died 3 days later. In this article we discussed about sunitinib-mediated inhibition of adenosine monophosphate kinase (AMPK) as a possible pathophysiology of rhabdomyolysis. Our case is the third sunitinib-induced rhabdomyolysis reported in the literature.

## 1. Introduction

Kidney cancer is among the ten most frequently diagnosed cancers in men and women in the United States, with more than an estimated 62,000 new cases in 2016. The prognosis has historically been poor, with current 5-year survival rates of 74% overall, decreasing to 53% among patients with locoregional (stage III) disease and 8% among patients with metastatic disease. Although more than 14,000 patients die from kidney cancer each year, there has been considerable progress in the systemic treatment of metastatic RCC in the past 20 years. Clear cell RCC makes up approximately 70% of RCCs [1].

In 2005 and 2006, the Food and Drug Administration (FDA) approved multikinase inhibitors sorafenib and sunitinib, respectively. The approval of five other antiangiogenic drugs (pazopanib, axitinib, bevacizumab, cabozantinib, and lenvatinib) followed. Two mTOR inhibitors, temsirolimus and everolimus, were approved in later years. The immune checkpoint inhibitor nivolumab showed benefit in randomized phase 3 trials and was approved by the FDA in 2015. Sunitinib is an oral multitargeted drug against the VEGF receptors (VEGFRs) 1, 2, and 3; platelet-derived growth factor receptors (PDGFRs); and other tyrosine kinases. Sunitinib has been associated with higher response rate, longer progression-free survival, and overall survival than interferon alfa [1].

## 2. Case Presentation

The patient is a 71-year-old white male who was found to have a 3.5 cm right kidney mass and had been followed by the urology team closely at VA Pittsburgh Healthcare System. Urine cytology was suspicious for malignant cells. He underwent a radical right nephrectomy on February 3, 2014. Pathology showed clear cell RCC. The tumor was located at the lower pole with a size of 4.5 cm (pT1b) and Fuhrman nuclear grade 2. All margins were not involved by carcinoma, and there was no vascular invasion. He had been followed with a regular CT scan every year. He was found to have small bilateral lung metastasis and lymphadenopathy in 2016. The PET scan on April 26, 2016, revealed FDG activity in the lung and hilar and mediastinal lymph nodes. He underwent endobronchial ultrasound biopsy of the mediastinal lymph node which confirmed to be metastatic from clear cell RCC. Due to his comorbidities and mild thrombocytopenia, we started him on lower dose sunitinib at 37.5 mg per oral daily ×4 weeks every 6 weeks in May 2016. In total, he received 7 cycles of sunitinib. He had been followed every 6 weeks in the clinic. He only developed fatigue due to mild hypothyroidism for which he received levothyroxine. During the follow-up, he was found to have worsening thrombocytopenia with platelet counts in the range of 60,000 to 90,000. A follow-up CT scan and PET scan in October 2016 showed improvement of the lung metastasis and lymphadenopathy. He was last seen in the clinic on March 13, 2017.

He was admitted on March 29, 2017, due to muscle weakness, fatigue, poor oral intake, and difficulty swallowing for 2 weeks. During admission, his platelet count was found to be 13,000, serum creatinine 2.3, total bilirubin 4, AST/ALT > 2000, INR 2.9, calcium 7.5, creatine phosphokinase (CPK) > 5000, and uric acid 12 (see Table 1). Sunitinib was discontinued on the first day of admission. CT head revealed no evidence of metastatic disease. Chest X-ray did not show evidence of infiltration or effusion. Echocardiogram showed severe global hypokinesia with LVEF of 30–35%. His LVEF was 55% prior to starting on sunitinib. He quickly developed lactic acidosis and acute respiratory failure. In the intensive care unit, he received bicarbonate, high-dose oxygen, furosemide, and treatment for hyperkalemia. Despite all treatment support, he continued to decline. His family chose to deescalate care, and he died on April 1, 2017.

## 3. Discussion

Sunitinib is one of the standards of care therapy in patients with metastatic RCC with good or intermediate risk factors based on Memorial Sloan Kettering Cancer Center (MSKCC) risk factor criteria. Our patient had achieved a good response in the lung metastasis and hilar and mediastinal lymphadenopathy; however, after 10 months on sunitinib, he was admitted with anemia, worsening thrombocytopenia, hyperuricemia, acute renal failure, and evidence of myocardial failure. He was found to have severe global myocardial hypokinesia with LVEF decreased from 55% prior to sunitinib to 30% to 35% after initiating treatment with sunitinib. The diagnosis of sunitinib-induced rhabdomyolysis is based on the fact that he developed multiorgan failure and cardiomyopathy with CPK increased to more than 5000. Sunitinib is the only plausible cause to his clinical presentation. Ruggeri et al. reported two cases of rhabdomyolysis while on sunitinib in metastatic RCC [2]. Both patients developed exactly the same clinical and laboratory abnormalities like our patient with high AST/ALT and very high CPK. One patient died three days later, and the other patient survived after emergent hemodialysis for his anuria [2].

Side effects of sunitinib include, but are not limited to, fatigue, nausea, diarrhea, hypertension, hypothyroidism, cardiac toxicity, and skin toxicity. A decrease in the LVEF is one of the main cardiac toxicities of sunitinib. Grade 3 LVEF decline was reported in 3% in phase III first-line clinical sunitinib trial [3, 4]. Sunitinib inhibits VEGFRs 1, 2, and 3; PDGFRs, and other tyrosine kinases. PDGFRs, which are expressed in cardiomyocytes, have been reported to serve a protective role in the heart exposed to ischemic injury. However, the study by Edelberg et al. employed exogenous administration of PDGF to the heart [5]. But it was unclear whether the inhibition of endogenous PDGFRs by sunitinib would induce cardiotoxicity.

Kerkela et al. obtained an endomyocardial biopsy in a patient with RCC who developed acute decompensated systolic heart failure after 11 months of sunitinib treatment (37.5 mg daily dose) [6]. Prior to therapy, the LVEF was 65%. When the patient presented with heart failure, the LVEF had decreased to 20–25%. Transmission electron microscopy revealed widespread and severe structural alterations in mitochondria which included markedly swollen mitochondria. The authors identified in the human heart biopsy that off-target inhibition by sunitinib demonstrated disruption of the mitochondrial architecture and an IC50 for adenosine monophosphate kinase (AMPK) inhibition as low as 0.2 mM [6].

**Figure 1:**
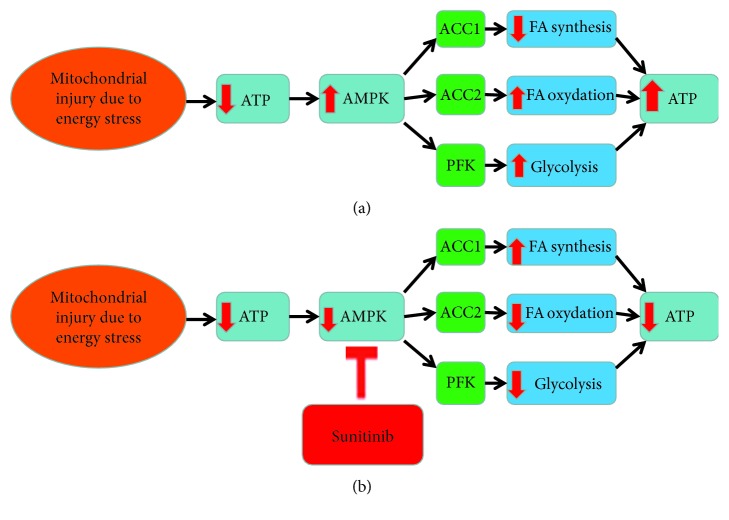
(a) Activation of AMP-kinase leads to the increase in ATP. Energy stress causes a decrease in ATP and in turn leads to activation of AMP-kinase. This will induce energy-generating pathways by rapid phosphorylation of acetyl coA carboxylases 1 and 2 (ACC1 and ACC2) and phosphofructokinase (PFK), which lead to decreased fatty acid synthesis, increased fatty acid oxidation, and increased glycolysis. This mechanism is to restore energy homeostasis. (b) Inhibition of AMP-kinase by sunitinib leads to a decrease in ATP. In the presence of sunitinib, ATP cannot bind to AMPK. This will prevent the activation of energy generating pathways and exacerbate energy-consuming pathways.

AMPK is a critical kinase in times of energy depletion when adenosine triphosphate (ATP) levels decline and adenosine monophosphate (AMP) levels increase. AMPK activation blocks energy-consuming pathways, including protein translation/synthesis and fatty acid synthesis. AMPK activates energy-generating pathways by increasing fatty acid oxidation via phosphorylation of acetyl-coA carboxylase 2 (ACC2) and glycolysis via activation of phosphofructo-2-kinase (PFK) (Figure 1(a)). Sunitinib-mediated inhibition of AMPK could release these energy-consuming pathways and prevent activation of energy-generating pathways, exacerbating the energy rundown in the cell (Figure 1(b)) [6, 7]. It has been suggested that modification or reduction of sunitinib dose to no longer target AMPK might reduce cardiotoxicity.

**Figure 2:**
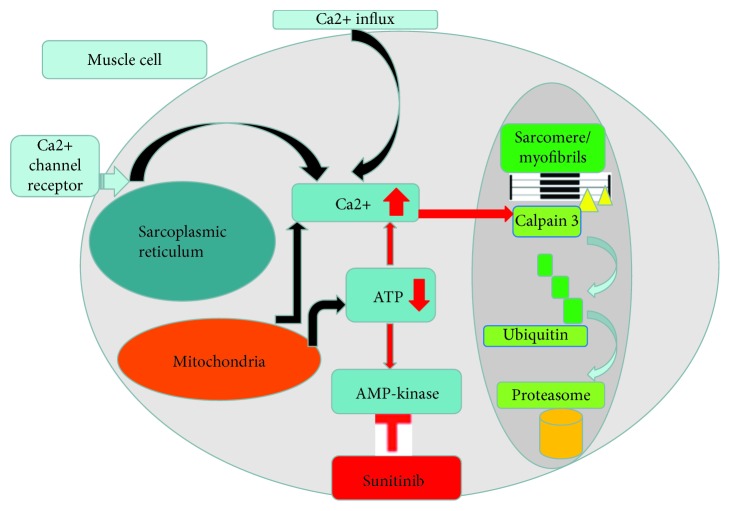
Sunitinib inhibits intracellular AMP-kinase and increase myoplasmic Ca2+. Accumulation of Ca2+ will activate calpain 3 kinase that in turn cause degradation of myofibrils. Ubiquitin ligases will ubiquitinate the peptides and target them for degradation in the proteasome.

Hohenegger also proposed the same mechanism that suggests sunitinib could interfere with fatty acid oxidation and glycolysis. Under stress, AMPK activity usually acts as a rescue pathway. Sunitinib inhibits AMPK thus causing a decline in intracellular ATP and elevation in myoplasmic Ca2+. Sufficient ATP supply by mitochondrial respiratory chain fails, and as a consequence, extrusion of Ca2+ to the extracellular space is reduced [8, 9]. Long-lasting Ca2+ elevations activate calpain proteases, which further degrade proteins that participate in Ca2+ homeostasis and thereby aggravate myoplasmic Ca2+ overload. The skeletal muscle-specific calpain 3 protease may contribute a further mechanism helping to explain the destruction of the myofibrils [10]. Calpain 3 is anchored to the sarcomere which performs the initial proteolytic cleavage that allows ubiquitin ligases to ubiquitinate the peptides and target them for degradation in the proteasome (Figure 2) [11].

Severe global myocardial hypokinesia and increase in CPK due to destruction in myofibrils are the most important clinical presentation of sunitinib-induced rhabdomyolysis. As reported in the two other cases by Ruggeri et al., CPK could be as high as 3000 to 5000. Myoglobin can be found in urine, but with a short half-life (about two to three hours). Serum CPK is a better marker for the diagnosis of rhabdomyolysis. CPK is active in skeletal muscles (CPK-MM) and catalyzes the transportation of one phosphate group from creatinine (C) to ADP, resulting in ATP. CPK is elevated in the first 12 hours after the onset of rhabdomyolysis, peaks within the first three days, and returns to the baseline level at three to five days after the injury. The half-life of CPK is 1.5 days. Therefore, CPK is a more reliable marker than myoglobin in the diagnosis of muscular damage [12].

## 4. Conclusion

Sunitinib-induced rhabdomyolysis is a very rare occurrence. Rhabdomyolysis presents with rapid deterioration of clinical condition due to liver failure, kidney failure, global hypokinesia of myocardium, very high CPK, hyperuricemia, and lactic acidosis that could lead to death. Inhibition of AMPK has been reported as the possible pathophysiology of this toxicity. Initial recognition of this toxicity is critical to ensure timely management.

## Figures and Tables

**Table 1:** 

LAB 2017	Day 1 (3/29)	Day 2 (3/30)	Day 3 (3/31)	Day 4 (4/1)
WBC (K/cmm)	5.4	6.4	7.5	5.3
HGB (g/dL)	14.7	14	14	12.6
Platelet (K/cmm)	13	17	18	13
Potassium (mmol/L)	5.8	6.1	4.8	4.0
AST (IU/L)	462	1984	2032	2450
ALT (IU/L)	351	1579	2277	2197
BUN (mg/dL)	29	30	31	31
Creatinine (mg/dL)	2.0	2.0	2.3	2.2
Bilirubin (mg/dL)	4.1	4.7	4.0	4.0
Uric acid (mg/dL)	—	12.1	—	—
Lactic acid (mmol/L)	—	—	9.4	10.5
CPK (IU/L)	1393	3667	5149	3582
Troponin (ng/mL)	0.10	0.11	0.12	—
